# Generalizability of an Automatic Explanation Method for Machine Learning Prediction Results on Asthma-Related Hospital Visits in Patients With Asthma: Quantitative Analysis

**DOI:** 10.2196/24153

**Published:** 2021-04-15

**Authors:** Gang Luo, Claudia L Nau, William W Crawford, Michael Schatz, Robert S Zeiger, Corinna Koebnick

**Affiliations:** 1 Department of Biomedical Informatics and Medical Education University of Washington Seattle, WA United States; 2 Department of Research & Evaluation Kaiser Permanente Southern California Pasadena, CA United States; 3 Department of Allergy and Immunology Kaiser Permanente South Bay Medical Center Harbor City, CA United States; 4 Department of Allergy Kaiser Permanente Southern California San Diego, CA United States

**Keywords:** asthma, forecasting, patient care management, machine learning

## Abstract

**Background:**

Asthma exerts a substantial burden on patients and health care systems. To facilitate preventive care for asthma management and improve patient outcomes, we recently developed two machine learning models, one on Intermountain Healthcare data and the other on Kaiser Permanente Southern California (KPSC) data, to forecast asthma-related hospital visits, including emergency department visits and hospitalizations, in the succeeding 12 months among patients with asthma. As is typical for machine learning approaches, these two models do not explain their forecasting results. To address the interpretability issue of black-box models, we designed an automatic method to offer rule format explanations for the forecasting results of any machine learning model on imbalanced tabular data and to suggest customized interventions with no accuracy loss. Our method worked well for explaining the forecasting results of our Intermountain Healthcare model, but its generalizability to other health care systems remains unknown.

**Objective:**

The objective of this study is to evaluate the generalizability of our automatic explanation method to KPSC for forecasting asthma-related hospital visits.

**Methods:**

Through a secondary analysis of 987,506 data instances from 2012 to 2017 at KPSC, we used our method to explain the forecasting results of our KPSC model and to suggest customized interventions. The patient cohort covered a random sample of 70% of patients with asthma who had a KPSC health plan for any period between 2015 and 2018.

**Results:**

Our method explained the forecasting results for 97.57% (2204/2259) of the patients with asthma who were correctly forecasted to undergo asthma-related hospital visits in the succeeding 12 months.

**Conclusions:**

For forecasting asthma-related hospital visits, our automatic explanation method exhibited an acceptable generalizability to KPSC.

**International Registered Report Identifier (IRRID):**

RR2-10.2196/resprot.5039

## Introduction

### Background

Asthma affects 8.4% of the US population [1], resulting in over 3000 deaths, almost 500,000 hospitalizations, and over 2,000,000 emergency department (ED) visits every year [1,2]. The state-of-the-art method for reducing asthma-related hospital visits, including ED visits and hospitalizations, is to use a model to forecast which of the patients with asthma are prone to future poor outcomes. We then enroll these patients in care management, ensuring care managers call them periodically to help them schedule health and related services. As used by health care systems like University of Washington Medicine, Intermountain Healthcare, and Kaiser Permanente Northern California [3] and by health plans in 9 of 12 metropolitan communities [4], this method, if implemented properly, can cut up to 40% of patients’ future hospital visits [5-8].

Having a limited capacity, a care management program can enroll only a small portion of patients [9], and its effectiveness is upper bounded by the accuracy of the predictive model. Because they are missing certain important features, the existing models for forecasting asthma-related hospital visits among patients with asthma [3,10-22] are inaccurate, with each model missing over half of patients who will undergo future asthma-related hospital visits and mislabeling many others as making such visits. As a result, care management programs continue to be used inefficiently because they are unable to focus on the highest-risk patients. In addition, patient outcomes deteriorate, whereas health care costs increase. To address this problem, we recently considered many candidate features and developed two extreme gradient boosting (XGBoost) [23] machine learning models, one on Intermountain Healthcare data [24] and the other on Kaiser Permanente Southern California (KPSC) data [25], to forecast asthma-related hospital visits in the succeeding 12 months among patients with asthma with a higher accuracy. As is typical for machine learning approaches, these two models do not explain their forecasting results. Clinicians would know that a patient is considered to be at a high risk by the model, but the model offers no reason why this is the case. This makes it difficult for clinicians to understand and trust the model’s prediction result, determine whether the patient should be put into care management, and pinpoint interventions suitable for the patient. To address the interpretability issue of black-box models, we designed an automatic method to offer rule format explanations for any machine learning model’s forecasting results on imbalanced tabular data and to suggest customized interventions with no accuracy loss [26]. Our method worked well for explaining our Intermountain Healthcare model’s forecasting results [26], but its generalizability to other health care systems remains unknown.

### Objectives

The objective of this study is to evaluate the generalizability of our automatic explanation method to KPSC in forecasting asthma-related hospital visits. In the following sections, we describe our evaluation approach and results.

## Methods

### Ethics Approval and Study Design

After receiving approval from the institutional review boards of KPSC and University of Washington Medicine, in this study, we conducted a secondary analysis of retrospective data.

### Patient Population

We adopted the same patient cohort from our previous KPSC predictive model paper [25]: a random sample of 70% of patients with asthma who had a KPSC health plan for any period between 2015 and 2018. This sample size is the largest one permitted by KPSC for sharing its data with another non-Kaiser Permanente institution for research. KPSC has 227 clinics and 15 hospitals. It is the largest integrated health care system in Southern California, offering care to approximately 19% of residents there [27]. A patient was deemed asthmatic in a specific year if during that year, at least one asthma diagnosis code (*International Classification of Diseases, Tenth Revision* [*ICD-10*]: J45.x; *International Classification of Diseases, Ninth Revision* [*ICD-9*]: 493.0x, 493.1x, 493.8x, and 493.9x) was recorded on the patient in the encounter billing database [11,28,29]. Patient death during that year served as the exclusion criterion.

### Prediction Target (Dependent Variable)

We adopted the same prediction target as our prior KPSC predictive model paper [25]. For every patient deemed to have asthma in a specific year, the indicator of any asthma-related hospital visit in the succeeding year is the outcome. An asthma-related hospital visit is a hospitalization or ED visit with asthma as its principal diagnosis (*ICD-10*: J45.x; *ICD-9*: 493.0x, 493.1x, 493.8x, and 493.9x). When training and testing our automatic explanation method and our KPSC XGBoost model, for each patient who had a KPSC health plan on a year’s last day and was also deemed asthmatic in the year, we used the patient’s data up to the year’s last day to forecast the patient’s outcome in the succeeding year.

### Data Set

We adopted the same administrative and clinical data set from our prior KPSC predictive model paper [25]. Obtained from KPSC’s research data warehouse, this structured data set covered our patient cohort’s visits at KPSC between 2010 and 2018.

### Features (Independent Variables), Predictive Models, and Data Preprocessing

Our KPSC model [25] uses the XGBoost classification algorithm [23] and 221 features to forecast asthma-related hospital visits in the succeeding year in patients with asthma. These features are listed in our previous KPSC predictive model paper [25], were computed on the structured attributes in our data set, and cover various characteristics such as patient demographics, medications, visits, diagnoses, vital signs, procedures, and laboratory tests. An example feature is the total number of asthma relievers that the patient filled in the previous 12 months. Every input data instance to our KPSC model aims at a (patient, index year) pair, includes these 221 features, and is used to forecast the succeeding year’s outcome of the patient. As in our prior KPSC predictive model paper [25], the top 10% of patients with asthma projected at the highest risk were used as the cutoff point for binary classification. We used the same data preprocessing approach adopted in our prior KPSC predictive model paper [25] to clean, normalize, and prepare the data.

### Review of Our Automatic Explanation Method

Previously, we designed an automatic method to offer rule format explanations for the forecasting results of any machine learning model on tabular data and to suggest customized interventions with no accuracy loss. The original method [30] was designed for relatively balanced data. Recently, we extended the method to handle imbalanced data [26], where one value of the outcome variable has a much lower prevalence rate than another. This fits the case of forecasting asthma-related hospital visits in patients with asthma. At KPSC, the prevalence rate of having asthma-related hospital visits in the succeeding year was approximately 2%. In the remainder of this paper, we focus on the extended automatic explanation method.

#### Main Idea

The central idea of our automatic explanation method is to use two models side by side to separate the forecasting and offering explanations. Each model serves a different purpose. The first model was used for the forecasting. Typically chosen to be the most accurate one, this model can be any model built on continuous and categorical features. The second model contains class-based association rules [31,32] mined from past data. It is used not to forecast but to explain the forecasting results of the first model. After using an automatic discretization method [31,33] to convert continuous features to categorical features, we use a standard approach such as Apriori to mine the association rules [32]. Each rule presents a feature pattern linking to a value *u* of the outcome variable and has the form:

*r*_1_ AND *r*_2_ AND ... AND *r_s_* → *u*.

The values of *s* and *u* can differ across the rules. For the binary classification of poor versus good outcomes, *u* is typically a poor outcome value. Each item *r_i_* (1≤*i*≤*s*) is a feature-value pair (*g*, *v*). When *v* is a value, *r_i_* shows that feature *g* has a value *v*. When *v* is a range, *r_i_* indicates that the value of *g* is within *v*. The rule signifies that a patient’s outcome is apt to be *u* if *r*_1_, *r*_2_, ..., and *r_s_* are all satisfied by the patient. An exemplar rule is:

The patient had 8 or 9 primary or principal asthma diagnoses in the previous 12 months

AND the patient had ≥6 no shows in the prior 12 months

→ The patient will undergo ≥1 asthma-related hospital visit in the subsequent 12 months.

#### The Rule Mining and Pruning Process

Our automatic explanation method uses 5 parameters: the minimum commonality threshold, the minimum confidence threshold, the largest number of items permitted on an association rule’s left-hand side, the confidence difference threshold, and the number of top features used to construct rules. For a given rule

*r_1_* AND *r_2_* AND ... AND *r_s_* → *u*,

its commonality reflects its coverage in the context of *u* and refers to the fraction of data instances fulfilling *r*_1_, *r*_2_, ..., and *r_s_* among all the data instances connected to *u*. Its confidence reflects its precision and refers to the fraction of data instances connecting to *u* among all the data instances fulfilling *r*_1_, *r*_2_, ..., and *r_s_*. Our method uses those rules whose commonality is no less than the minimum commonality threshold, whose confidence is no less than the minimum confidence threshold, and each containing no more than the maximum permitted number of items on its left-hand side.

We use 3 techniques to reduce the number of association rules and prevent it from being excessively large. First, we remove every more specific rule *q*_1_ in the presence of a more general rule *q*_2_ satisfying *q*_2_’s confidence ≥ *q*_1_’s confidence−the confidence difference threshold. Second, certain machine learning algorithms, such as XGBoost [23], can automatically compute every feature’s importance value. When handling a large data set with many features, only the top few features having the largest importance values and used in the first model are adopted to construct rules. Third, a clinician in the design team of the automatic explanation function examines all possible values and value ranges of the features adopted to construct rules and labels those values and value ranges that could have a positive correlation with the poor outcome value. Only the labeled feature values and value ranges are adopted to form rules.

For each feature-value pair item that is adopted to construct association rules, a clinician in the design team of the automatic explanation function compiles zero or more interventions. We tag an item actionable if it links to at least one intervention. Each rule passing the rule pruning process is automatically linked to the interventions related to the actionable items on the left-hand side of the rule. We tag a rule actionable if it contains at least one actionable item on its left-hand side; that is, it links to at least one intervention.

#### The Explanation Approach

For every patient whom the first model forecasts to take a poor outcome value, we explain the forecasting result by showing the association rules in the second model having this value on their right-hand sides and whose left-hand sides are satisfied by the patient. Each rule provides a reason why the patient is forecasted to take this value. For each actionable rule that is shown, the interventions connected to it are listed next to it. The automatic explanation function’s user can find customized interventions that fit the patient from the listed interventions. Usually, the rules in the second model present common reasons for having poor outcomes. Some patients will experience poor outcomes for other reasons. Thus, the second model can explain most, but not all, of the poor outcomes correctly forecasted by the first model.

### Parameter Setting

In our experiments, we used the same parameter setting approach used in our previous automatic explanation paper [26]. Each association rule had no more than 5 items on its left-hand side. Our KPSC XGBoost model [25] used 221 features to forecast asthma-related hospital visits. We used the top 50 features that our KPSC model ranked with the largest importance values to construct association rules. Our KPSC model gained an area under the receiver operating characteristic curve (AUC) of 0.820 using all 221 features and an AUC of 0.815 using the top 50 features.

For forecasting asthma-related hospital visits, our KPSC model [25] obtained a lower AUC for KPSC data than our Intermountain Healthcare model on Intermountain Healthcare data [24]. As mentioned in our previous automatic explanation paper [26], the harder it is to forecast the outcome, the smaller the minimum commonality and confidence thresholds need to be to ensure that our automatic explanation method can provide explanations for a large percentage of the patients whom the first model correctly forecasts to take a poor outcome value. Following this guideline on KPSC data, we set the minimum commonality threshold to 0.08%, which is lower than the corresponding value of 0.2% we used on Intermountain Healthcare data [26]. We set the minimum confidence threshold to 25%, which is lower than the corresponding value of 50% used for the Intermountain Healthcare data [26]. Despite not looking large, 25% is much greater than 2%, which is the percentage of KPSC data instances associated with asthma-related hospital visits in the succeeding year, as well as our KPSC model’s positive predictive value of 11.03% [25].

To set the value of the confidence difference threshold *τ*, we calculated the number of association rules passing the rule pruning process versus *τ*. Our previous paper [26] shows that this number of rules first drops quickly as *τ* rises and then drops slowly when *τ* becomes sufficiently large. The value of *τ* was set at the transition point.

### Data Analysis

#### Partitioning of the Training and Test Sets

We used the same method adopted in our prior KPSC predictive model paper [25] to divide the entire data set into training and test sets. As several features were computed on the data from up to 2 years before the index year and the outcomes came from the succeeding year, our data set included 6 years of effective data (2012-2017) over the 9-year period of 2010-2018. To match the use of our KPSC model and our automatic explanation method in clinical practice, we used the 2012-2016 data as the training set to train our KPSC model and mine the association rules adopted by our automatic explanation method. We used the 2017 data as the test set to gauge the performance of our KPSC model and the automatic explanation method.

#### Performance Metrics

We used the same performance metrics from our previous automatic explanation paper [26] to assess the performance of our automatic explanation method. A performance metric on our method’s explanation power is the fraction of patients with asthma whom our method could offer explanations for among the patients whom our KPSC model correctly forecasted to undergo asthma-related hospital visits in the succeeding year. We computed the average number of rules and the average number of actionable rules that suit such a patient. A rule suits a patient if all items on its left-hand side are satisfied for the patient.

As our previous automatic explanation paper [26] showed, several rules suiting a patient often differ by a single item on their left-hand sides. When multiple rules suit a patient, the amount of nonredundant information included in them is usually much less than the number of rules in them. To plot a full picture of the amount of information included in the automatic explanations given to the patients, we computed 3 distributions of the patients with asthma whom our KPSC model correctly forecasted to undergo asthma-related hospital visits in the succeeding year: (1) by the number of actionable rules suiting a patient, (2) by the number of different actionable items included in all the rules suiting a patient, and (3) by the number of rules suiting a patient.

## Results

### Demographic and Clinical Characteristics of Our Patient Cohort

Remember that each data instance aims at a different (patient, index year) pair. Tables 1 and 2 present the demographic and clinical characteristics of our KPSC patient cohort during 2012-2016 and 2017, respectively. The two sets of characteristics are sufficiently similar to each other. During 2012-2016, 2.42% (18,925/782,762) of data instances were linked to asthma-related hospital visits in the succeeding year. During 2017, this fraction was 2.13% (4353/204,744). Our previous KPSC predictive model paper [25] provides a detailed comparison of the two sets of characteristics.

**Table 1:** Demographic and clinical characteristics of our Kaiser Permanente Southern California patient cohort during 2012-2016.

Characteristics			Data instances associated with no asthma-related hospital visit in the succeeding year (n=763,837), n (%)		Data instances associated with asthma-related hospital visits in the succeeding year (n=18,925), n (%)		Data instances (n=782,762), n (%)
**Age (years)**							
	≥65	108,662 (14.23)		2288 (12.09)		110,950 (14.17)	
	18-65	415,889 (54.45)		8557 (45.22)		424,446 (54.22)	
	6 to <18	188,583 (24.69)		5039 (26.63)		193,622 (24.74)	
	<6	50,703 (6.64)		3041 (16.07)		53,744 (6.87)	
**Gender**							
	Female	443,410 (58.05)		10,590 (55.96)		454,000 (58.00)	
	Male	320,427 (41.95)		8335 (44.04)		328,762 (42.00)	
**Race**							
	White	477,542 (62.52)		10,040 (53.05)		487,582 (62.29)	
	Native Hawaiian or other Pacific Islander	7692 (1.01)		230 (1.22)		7922 (1.01)	
	Black or African American	110,869 (14.51)		4982 (26.33)		115,851 (14.80)	
	Asian	68,781 (9.00)		1282 (6.77)		70,063 (8.95)	
	American Indian or Alaska native	3745 (0.49)		86 (0.45)		3831 (0.49)	
	Unknown or unreported	95,208 (12.46)		2305 (12.18)		97,513 (12.46)	
**Ethnicity**							
	Non-Hispanic	449,795 (58.89)		10,577 (55.89)		460,372 (58.81)	
	Hispanic	299,240 (39.18)		8131 (42.96)		307,371 (39.27)	
	Unknown or unreported	14,802 (1.94)		217 (1.15)		15,019 (1.92)	
**Insurance**							
	Self-paid plan	104,479 (13.68)		2224 (11.75)		106,703 (13.63)	
	Public	216,320 (28.32)		7469 (39.47)		223,789 (28.59)	
	High deductible plan	80,393 (10.52)		1426 (7.54)		81,819 (10.45)	
	Exchange (also known as marketplace)	39,050 (5.11)		735 (3.88)		39,785 (5.08)	
	Commercial (employer-paid)	521,101 (68.22)		11,311 (59.77)		532,412 (68.02)	
	Other	265,264 (34.73)		6064 (32.04)		271,328 (34.66)	
**Number of years from the first visit related to** **asthma in the data set**							
	>3	439,930 (57.59)		10,919 (57.70)		450,849 (57.60)	
	≤3	323,907 (42.41)		8006 (42.30)		331,913 (42.40)	
**Asthma medication fill**							
	Systemic corticosteroid	236,246 (30.93)		10,837 (57.26)		247,083 (31.57)	
	Short-acting, inhaled beta-2 agonist	537,442 (70.36)		16,242 (85.82)		553,684 (70.73)	
	Mast cell stabilizer	20 (0.00)		0 (0.00)		20 (0.00)	
	Long-acting beta-2 agonist	33,576 (4.40)		1694 (8.95)		35,270 (4.51)	
	Leukotriene modifier	85,299 (11.17)		4125 (21.80)		89,424 (11.42)	
	Combination of long-acting beta-2 agonist and inhaled corticosteroid	88,847 (11.63)		3975 (21.00)		92,822 (11.86)	
	Inhaled corticosteroid	325,156 (42.57)		11,841 (62.57)		336,997 (43.05)	
**Comorbidity**							
	Sleep apnea	20,465 (2.68)		575 (3.04)		21,040 (2.69)	
	Sinusitis	112,341 (14.71)		2832 (14.96)		115,173 (14.71)	
	Premature birth	16,607 (2.17)		690 (3.65)		17,297 (2.21)	
	Obesity	171,666 (22.47)		4776 (25.24)		176,442 (22.54)	
	Gastroesophageal reflux	101,180 (13.25)		2778 (14.68)		103,958 (13.28)	
	Eczema	82,425 (10.79)		2944 (15.56)		85,369 (10.91)	
	Cystic fibrosis	135 (0.02)		3 (0.02)		138 (0.02)	
	Chronic obstructive pulmonary disease	27,388 (3.59)		999 (5.28)		28,387 (3.63)	
	Bronchopulmonary dysplasia	241 (0.03)		22 (0.12)		263 (0.03)	
	Anxiety or depression	160,719 (21.04)		4231 (22.36)		164,950 (21.07)	
	Allergic rhinitis	164,036 (21.48)		4673 (24.69)		168,709 (21.55)	
**Smoking status**							
	Never smoker or unknown	477,263 (62.48)		11,885 (62.80)		489,148 (62.49)	
	Former smoker	133,456 (17.47)		2870 (15.17)		136,326 (17.42)	
	Current smoker	153,118 (20.05)		4170 (22.03)		157,288 (20.09)	

**Table 2:** Demographic and clinical characteristics of our Kaiser Permanente Southern California patient cohort in 2017.

Characteristics			Data instances associated with no asthma-related hospital visit in the succeeding year (n=200,391), n (%)		Data instances associated with asthma-related hospital visits in the succeeding year (n=4353), n (%)		Data instances (n=204,744), n (%)
**Age (years)**							
	≥65	35,342 (17.64)		679 (15.60)		36,021 (17.59)	
	18-65	109,969 (54.88)		2052 (47.14)		112,021 (54.71)	
	6 to <18	43,856 (21.89)		1012 (23.25)		44,868 (21.91)	
	<6	11,224 (5.60)		610 (14.01)		11,834 (5.78)	
**Gender**							
	Female	118,013 (58.89)		2482 (57.02)		120,495 (58.85)	
	Male	82,378 (41.11)		1871 (42.98)		84,249 (41.15)	
**Race**							
	White	124,514 (62.14)		2302 (52.88)		126,816 (61.94)	
	Native Hawaiian or other Pacific Islander	1910 (0.95)		42 (0.96)		1952 (0.95)	
	Black or African American	26,864 (13.41)		1075 (24.70)		27,939 (13.65)	
	Asian	18,555 (9.26)		319 (7.33)		18,874 (9.22)	
	American Indian or Alaska native	987 (0.49)		31 (0.71)		1018 (0.50)	
	Unknown or unreported	27,561 (13.75)		584 (13.42)		28,145 (13.75)	
**Ethnicity**							
	Non-Hispanic	116,801 (58.29)		2410 (55.36)		119,211 (58.22)	
	Hispanic	78,153 (39.00)		1868 (42.91)		80,021 (39.08)	
	Unknown or unreported	5437 (2.71)		75 (1.72)		5512 (2.69)	
**Insurance**							
	Self-paid plan	33,758 (16.85)		647 (14.86)		34,405 (16.80)	
	Public	64,727 (32.30)		1904 (43.74)		66,631 (32.54)	
	High deductible plan	24,647 (12.30)		356 (8.18)		25,003 (12.21)	
	Exchange (also known as marketplace)	17,677 (8.82)		269 (6.18)		17,946 (8.77)	
	Commercial (employer-paid)	127,724 (63.74)		2420 (55.59)		130,144 (63.56)	
	Other	83,108 (41.47)		1675 (38.48)		84,783 (41.41)	
**Number of years from the first visit related to** **asthma in the data set**							
	>3	116,285 (58.03)		2616 (60.10)		118,901 (58.07)	
	≤3	84,106 (41.97)		1737 (39.90)		85,843 (41.93)	
**Asthma medication fill**							
	Systemic corticosteroid	64,878 (32.38)		2597 (59.66)		67,475 (32.96)	
	Short-acting, inhaled beta-2 agonist	137,077 (68.40)		3742 (85.96)		140,819 (68.78)	
	Mast cell stabilizer	0 (0.00)		0 (0.00)		0 (0.00)	
	Long-acting beta-2 agonist	11,343 (5.66)		467 (10.73)		11,810 (5.77)	
	Leukotriene modifier	26,996 (13.47)		1099 (25.25)		28,095 (13.72)	
	Combination of long-acting beta-2 agonist and inhaled corticosteroid	28,580 (14.26)		1151 (26.44)		29,731 (14.52)	
	Inhaled corticosteroid	78,220 (39.03)		2586 (59.41)		80,806 (39.47)	
**Comorbidity**							
	Sleep apnea	12,811 (6.39)		333 (7.65)		13,144 (6.42)	
	Sinusitis	29,202 (14.57)		680 (15.62)		29,882 (14.59)	
	Premature birth	4381 (2.19)		132 (3.03)		4513 (2.20)	
	Obesity	48,548 (24.23)		1190 (27.34)		49,738 (24.29)	
	Gastroesophageal reflux	32,462 (16.20)		797 (18.31)		33,259 (16.24)	
	Eczema	20,521 (10.24)		638 (14.66)		21,159 (10.33)	
	Cystic fibrosis	40 (0.02)		2 (0.05)		42 (0.02)	
	Chronic obstructive pulmonary disease	7306 (3.65)		285 (6.55)		7591 (3.71)	
	Bronchopulmonary dysplasia	29 (0.01)		1 (0.02)		30 (0.01)	
	Anxiety or depression	46,176 (23.04)		1124 (25.82)		47,300 (23.10)	
	Allergic rhinitis	39,849 (19.89)		1084 (24.90)		40,933 (19.99)	
**Smoking status**							
	Never smoker or unknown	125,245 (62.50)		2663 (61.18)		127,908 (62.47)	
	Former smoker	36,026 (17.98)		717 (16.47)		36,743 (17.95)	
	Current smoker	39,120 (19.52)		973 (22.35)		40,093 (19.58)	

### The Number of Residual Association Rules

**Figure 1:**
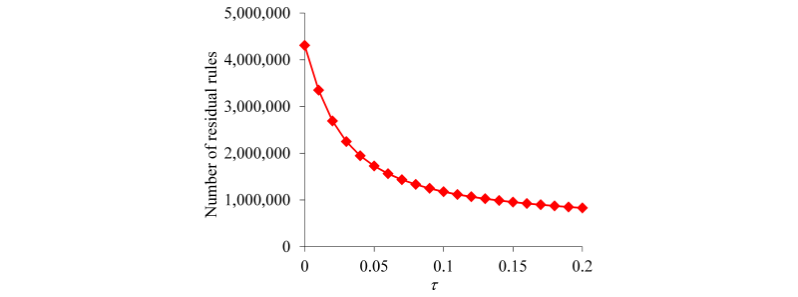
The number of residual rules versus the confidence difference threshold *τ*.

Taking the top 50 features that our KPSC model ranked with the largest importance values, we mined 11,628,850 association rules from the training set. Figure 1 displays the number of residual rules versus the confidence difference threshold *τ*. This number first drops quickly as *τ* rises and then drops slowly when *τ* becomes ≥0.15. Accordingly, the value of *τ* was set to 0.15, resulting in 954,493 residual rules.

An asthma clinical expert in our team labeled the values and value ranges of the top 50 features that could have a positive correlation with asthma-related hospital visits in the succeeding year. After we removed those rules involving any other value or value range, 725,632 association rules remained. Each rule provides a reason why a patient is forecasted to undergo future asthma-related hospital visits. Almost all (725,623) of these rules were actionable. Thus, our automatic explanation method’s performance numbers are almost the same regardless of whether all these rules or only the actionable rules were used. In the remainder of this section, we present only the performance numbers when only the actionable rules were used.

### Example Association Rules Adopted by the Second Model

To allow the reader to gain a sense of the association rules the second model adopted, we present 5 example rules:

- Rule 1: The patient filled ≥89 asthma relievers in total in the previous 12 months

→ The patient will undergo ≥1 asthma-related hospital visits in the subsequent 12 months.

The use of many asthma relievers indicates poor asthma control. An intervention tied to the item *the patient filled ≥89 asthma relievers in total in the prior 12 months* is to tailor prescribed medications and to suggest the patient to maximize adherence to asthma control medications or to improve avoidance of asthma triggers.

- Rule 2: The patient had ≥25 nebulizer medication orders in the previous 12 months

AND the patient incurred ≥16 major visits for asthma in the previous 12 months

→ The patient will undergo ≥1 asthma-related hospital visits in the subsequent 12 months.

The use of many nebulizer medications indicates a poor asthma control. An intervention tied to the item *the patient had ≥25 nebulizer medication orders in the prior 12 months* is to tailor prescribed medications and to suggest the patient to maximize adherence to asthma control medications or to improve the avoidance of asthma triggers.

As defined in our previous paper [24], major visits for asthma cover outpatient visits linked to a primary diagnosis of asthma, and ED visits and hospitalizations linked to an asthma diagnosis code. Outpatient visits linked to a secondary, but not a primary, diagnosis of asthma are deemed minor visits for asthma. Having many major visits for asthma indicates a poor asthma control. An intervention tied to the item *the patient incurred ≥16 major visits for asthma in the prior 12 months* is to adopt control strategies for the patient to avoid needing emergency care.

- Rule 3: The patient had 8 or 9 primary or principal asthma diagnoses in the previous 12 months

AND the patient had ≥6 no shows in the previous 12 months

→ The patient will undergo ≥1 asthma-related hospital visits in the subsequent 12 months.

Having many primary or principal asthma diagnoses indicates a poor asthma control. An intervention tied to the item *the patient had 8 or 9 primary or principal asthma diagnoses in the prior 12 months* is to offer the patient suggestions on how to improve asthma control.

Having many no shows correlates with poor outcomes. An intervention tied to the item *the patient had ≥6 no shows in the prior 12 months* is to give the patient social resources to handle socioeconomic challenges to keep appointments.

- Rule 4: The patient incurred ≥8 ED visits in the previous 12 months

AND the patient was prescribed ≥28 short-acting beta-2 agonist medications in total in the previous 12 months

AND the patient is Black or African American

→ The patient will undergo ≥1 asthma-related hospital visits in the subsequent 12 months.

In the United States, Black and African American people tend to have poorer asthma outcomes than others. Frequent ED visits indicated a poor asthma control. An intervention tied to the item *the patient incurred ≥8 ED visits in the prior 12 months* is to adopt control strategies for the patient to avoid needing emergency care.

Short-acting beta-2 agonists are rescue medications for the quick relief of asthma symptoms. The use of many short-acting beta-2 agonists indicates a poor asthma control. An intervention tied to the item *the patient was ordered ≥28 short-acting beta-2 agonist medications in total in the prior 12 months* is to tailor prescribed medications and to suggest the patient to maximize adherence to asthma control medications or to improve the avoidance of asthma triggers.

- Rule 5: The highest exacerbation severity of all asthma diagnoses recorded on the patient in the previous 12 months is status asthmaticus

AND the patient incurred ≥11 and ≤17 visits with same-day appointments in the previous 12 months

AND the admission type of the patient’s last visit in the previous 12 months is nonelective

→ The patient will undergo ≥1 asthma-related hospital visits in the subsequent 12 months.

Status asthmaticus is the most severe form of asthma exacerbation. An intervention tied to the item *the highest exacerbation severity of all of the asthma diagnoses recorded on the patient in the prior 12 months is status asthmaticus* is to offer the patient suggestions on how to improve asthma control.

Having many visits with same-day appointments indicates a poor asthma control. An intervention tied to the item *the patient incurred ≥11 and ≤17 visits with same day appointments in the prior 12 months* is to improve support offered to the patient between visits to enhance medication adherence, address asthma triggers, and maximize the value of each visit.

A patient incurs a nonelective visit when the patient’s condition requires an immediate medical attention, for example, when the patient experiences severe asthma exacerbation. An intervention tied to the item *the admission type of the patient’s last visit in the prior 12 months is nonelective* is to adopt control strategies for the patient to avoid needing emergency care.

### The Performance of Our Automatic Explanation Method

We evaluated our automatic explanation method on the test set. Our method explained the forecasting results for 97.9% (599/612) of the children (age <18 years) with asthma and 97.45% (1605/1647) of the adults (age ≥18 years) with asthma our KPSC model correctly forecasted to undergo asthma-related hospital visits in the succeeding year. Put together, our method explained the forecasting results for 97.57% (2204/2259) of the patients with asthma who were correctly forecasted to undergo asthma-related hospital visits in the succeeding year. For every such patient, on average, our method provided 1516.25 (SD 2161.30) explanations, each from one rule, and found 24.04 (SD 8.68) actionable items.

**Figure 2:**
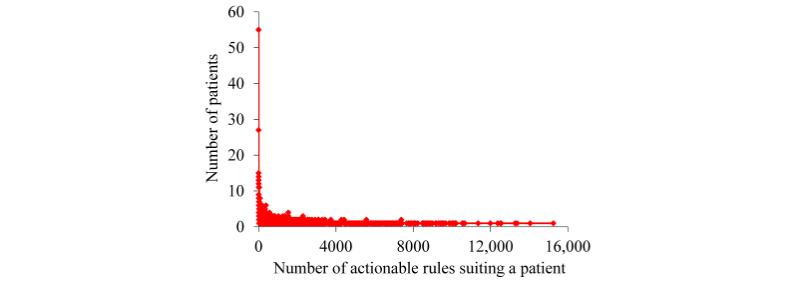
The patient distribution by the number of actionable rules suiting a patient for the patients with asthma whom our Kaiser Permanente Southern California model correctly forecasted to undergo asthma-related hospital visits in the succeeding year.

**Figure 3:**
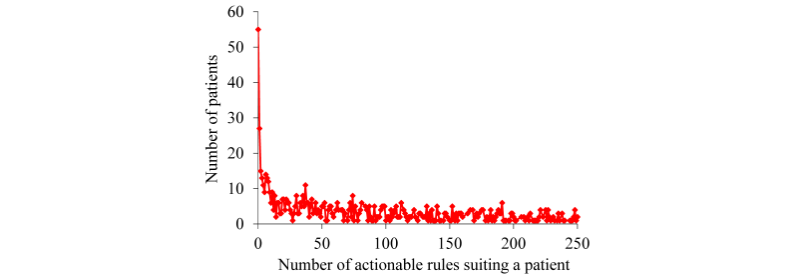
The patient distribution by the number of actionable rules suiting a patient when this number is ≤250 for the patients with asthma whom our Kaiser Permanente Southern California model correctly forecasted to undergo asthma-related hospital visits in the succeeding year.

For the patients with asthma whom our KPSC model correctly forecasted to undergo asthma-related hospital visits in the succeeding year, Figures 2 and 3 display the patient distribution according to the number of actionable rules suiting a patient. Having a long tail, this distribution is significantly skewed toward the left. As the number of rules suiting a patient increases, the number of patients each covered by this number of rules tends to decline nonmonotonically. The biggest number of rules suiting a patient is fairly large (15,252). However, only one patient matched this number of rules.

**Figure 4:**
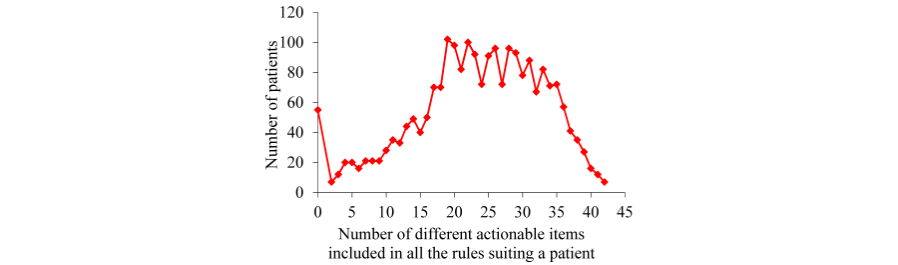
The patient distribution by the number of different actionable items included in all the rules suiting a patient for the patients with asthma whom our Kaiser Permanente Southern California model correctly forecasted to undergo asthma-related hospital visits in the succeeding year.

For the patients with asthma whom our KPSC model correctly forecasted to undergo asthma-related hospital visits in the succeeding year, Figure 4 displays the patient distribution according to the number of different actionable items included in all the rules suiting a patient. The largest number of different actionable items included in all the rules suiting a patient is 42, much less than the largest number of actionable rules suiting a patient. As noted in our previous automatic explanation paper [26], 2 or more actionable items included in the rules that suit a patient often connect to the same intervention.

Our automatic explanation method provided explanations for 67.61% (2943/4353) of patients with asthma who would undergo asthma-related hospital visits in the succeeding year.

## Discussion

### Principal Findings

The results presented in this paper are similar to those presented in our previous automatic explanation paper [26]. For forecasting asthma-related hospital visits, our automatic explanation method exhibited an acceptable generalizability to KPSC. In particular, our method explained the forecasting results for 97.57% (2204/2259) of the patients with asthma who were correctly forecasted to undergo asthma-related hospital visits in the succeeding year. This fraction is comparable with that (89.68%) on Intermountain Healthcare data in our previous automatic explanation paper [26] and is large enough to put our automatic explanation method into daily clinical use. After further development to boost its accuracy, our KPSC model combined with our automatic explanation method could be used to guide asthma care management’s use to help enhance patient outcomes and reduce health care costs.

Our automatic explanation method provided explanations for 67.61% (2943/4353) of patients with asthma who would undergo asthma-related hospital visits in the succeeding year. This fraction is less than the 97.57% (2204/2259) success rate, at which our method explained the forecasting results for the patients with asthma our KPSC model correctly forecasted to undergo asthma-related hospital visits in the succeeding year. This is possibly due to the correlation between the association rules’ and our KPSC model’s forecasting results. Among the patients with asthma whom our KPSC model correctly forecasted to undergo asthma-related hospital visits in the succeeding year, many are easy cases for us to explain their outcomes using association rules. Among the patients with asthma who would undergo asthma-related hospital visits in the succeeding year and whose outcomes were incorrectly forecasted by our KPSC model, many are difficult cases for any model to correctly explain or forecast their outcomes.

### Displaying the Automatic Explanations

Many rules could suit a patient. In this case, it is undesirable to list all of them simultaneously and overwhelm the user of the automatic explanation function. Instead, we should rank these rules and display the top few (eg, 3) of them by default. If desired, the user can ask an automatic explanation function to show more rules. In ranking the rules suiting a patient and the items on the left-hand side of a rule, we consider the following factors and strike a balance among them:

1. All else being equal, rules with fewer items on their left-hand sides are easier to understand and should be ranked higher.
2. All else being equal, rules with higher confidence are more precise and should be ranked higher.
3. All else being equal, rules with a higher commonality cover more patients with poor outcomes and should be ranked higher.
4. The automatic explanation function’s user tends to read the rules one by one in the display order. All else being equal, the more items on the left-hand side of a rule appear in higher-ranked rules, the less new information that the user has not seen so far is contained in the rule and the lower the rule should be ranked.
5. Consider the items on the left-hand side of a rule. The automatic explanation function’s user tends to read the items one by one in the display order. All else being equal, the items that have appeared in higher-ranked rules contain repeated information and should be put after the other items that have not appeared in any of the higher-ranked rules.
6. The automatic explanation function’s user cares about finding suitable interventions for the patient. Consider the items on the left-hand side of a rule. All else being equal, the actionable items should be placed before the nonactionable items.
7. Actionable rules should be ranked higher than nonactionable rules.

We are in the process of preparing a paper describing our rule-ranking method in detail.

### Related Work

As described in the book [34] and the survey paper [35], many other researchers have proposed miscellaneous methods for automatically offering explanations for the forecasting results of machine learning models. Such explanations are typically not in a rule format. Many such methods sacrifice a part of the forecasting accuracy and/or are designed for a particular machine learning algorithm. In addition, none of these methods can automatically suggest customized interventions. In comparison, our automatic explanation method supplies rule format explanations for any machine learning model’s forecasting results on tabular data and suggests customized interventions with no accuracy loss. Rule format explanations are easier to comprehend and can suggest customized interventions more directly than other forms of explanations.

To the best of our knowledge, we were the first to use association rules to automatically offer rule format explanations for any machine learning model’s forecasting results on tabular data and to suggest customized interventions with no accuracy loss [30]. Our original method [30] was designed for relatively balanced data and was initially tested in the case of forecasting type 2 diabetes diagnoses. Subsequently, Alaa et al [36,37] applied the original method to multiple medical prediction tasks. So far, no researcher outside of our group has applied our extended automatic explanation method [26], which can handle imbalanced data, to any prediction task. Rudin et al [38] and Ribeiro et al [39] used rules to automatically offer explanations for the forecasting results of any machine learning model. These rules are not association rules and are unknown before the prediction time; hence, they cannot be used to automatically suggest customized interventions at the prediction time. In comparison, the association rules used in our automatic explanation method are mined before the prediction time and used to automatically suggest customized interventions at the prediction time.

### Limitations

This study has three limitations, all of which can be fine areas for future work:

1. For forecasting asthma-related hospital visits, our study evaluated the generalizability of our automatic explanation method to a single health care system. It would be nice to assess our automatic explanation method’s generalizability to other health care systems, such as academic ones, which have different properties from Intermountain Healthcare and KPSC. In comparison with nonacademic systems, academic health care systems tend to handle more complex and sicker patients [40]. To prepare for such an evaluation, we are currently retrieving a data set of patients with asthma from the enterprise data warehouse of the University of Washington Medicine [41].
2. Our study evaluated the generalizability of our automatic explanation method only for forecasting asthma-related hospital visits. It would be nice to assess the generalizability of our automatic explanation method for other diseases and outcomes [41].
3. Our current automatic explanation method is designed for structured data and traditional machine learning algorithms that are not deep learning algorithms. It would be nice to extend our method so it can also handle deep learning models built directly on longitudinal data [41,42].

### Conclusions

In its first generalizability assessment, our automatic explanation method for imbalanced tabular data exhibited a decent generalizability to KPSC for forecasting asthma-related hospital visits. After further development to boost its accuracy, our KPSC model combined with our automatic explanation method could be used to guide asthma care management’s use to help enhance patient outcomes and reduce health care costs.

## Abbreviations

AUC: area under the receiver operating characteristic curve
ED: emergency department
ICD-9: International Classification of Diseases, Ninth Revision
ICD-10: International Classification of Diseases, Tenth Revision
KPSC: Kaiser Permanente Southern California
XGBoost: extreme gradient boosting

## References

[1] Moorman JE, Akinbami LJ, Bailey CM, Zahran HS, King ME, Johnson CA, Liu X (2012). National surveillance of asthma: United States, 2001-2010. Vital Health Stat 3.

[2] Nurmagambetov T, Kuwahara R, Garbe P (2018). The economic burden of asthma in the United States, 2008-2013. Ann Am Thorac Soc.

[3] Lieu TA, Quesenberry CP, Sorel ME, Mendoza GR, Leong AB (1998). Computer-based models to identify high-risk children with asthma. Am J Respir Crit Care Med.

[4] Mays GP, Claxton G, White J (2004). Managed care rebound? Recent changes in health plans' cost containment strategies. Health Aff (Millwood).

[5] Caloyeras JP, Liu H, Exum E, Broderick M, Mattke S (2014). Managing manifest diseases, but not health risks, saved PepsiCo money over seven years. Health Aff (Millwood).

[6] Greineder DK, Loane KC, Parks P (1999). A randomized controlled trial of a pediatric asthma outreach program. J Allergy Clin Immunol.

[7] Kelly CS, Morrow AL, Shults J, Nakas N, Strope GL, Adelman RD (2000). Outcomes evaluation of a comprehensive intervention program for asthmatic children enrolled in Medicaid. Pediatrics.

[8] Axelrod RC, Zimbro KS, Chetney RR, Sabol J, Ainsworth VJ (2001). A disease management program utilizing life coaches for children with asthma. J Clin Outcomes Manag.

[9] Axelrod RC, Vogel D (2003). Predictive modeling in health plans. Dis Manag Health Outcomes.

[10] Loymans RJB, Honkoop PJ, Termeer EH, Snoeck-Stroband JB, Assendelft WJJ, Schermer TRJ, Chung KF, Sousa AR, Sterk PJ, Reddel HK, Sont JK, Ter Riet G (2016). Identifying patients at risk for severe exacerbations of asthma: development and external validation of a multivariable prediction model. Thorax.

[11] Schatz M, Cook EF, Joshua A, Petitti D (2003). Risk factors for asthma hospitalizations in a managed care organization: development of a clinical prediction rule. Am J Manag Care.

[12] Eisner MD, Yegin A, Trzaskoma B (2012). Severity of asthma score predicts clinical outcomes in patients with moderate to severe persistent asthma. Chest.

[13] Sato R, Tomita K, Sano H, Ichihashi H, Yamagata S, Sano A, Yamagata T, Miyara T, Iwanaga T, Muraki M, Tohda Y (2009). The strategy for predicting future exacerbation of asthma using a combination of the Asthma Control Test and lung function test. J Asthma.

[14] Osborne ML, Pedula KL, O'Hollaren M, Ettinger KM, Stibolt T, Buist AS, Vollmer WM (2007). Assessing future need for acute care in adult asthmatics: the Profile of Asthma Risk Study: a prospective health maintenance organization-based study. Chest.

[15] Miller MK, Lee JH, Blanc PD, Pasta DJ, Gujrathi S, Barron H, Wenzel SE, Weiss ST, TENOR Study Group (2006). TENOR risk score predicts healthcare in adults with severe or difficult-to-treat asthma. Eur Respir J.

[16] Peters D, Chen C, Markson LE, Allen-Ramey FC, Vollmer WM (2006). Using an asthma control questionnaire and administrative data to predict health-care utilization. Chest.

[17] Yurk RA, Diette GB, Skinner EA, Dominici F, Clark RD, Steinwachs DM, Wu AW (2004). Predicting patient-reported asthma outcomes for adults in managed care. Am J Manag Care.

[18] Loymans RJ, Debray TP, Honkoop PJ, Termeer EH, Snoeck-Stroband JB, Schermer TR, Assendelft WJ, Timp M, Chung KF, Sousa AR, Sont JK, Sterk PJ, Reddel HK, Ter Riet G (2018). Exacerbations in adults with asthma: a systematic review and external validation of prediction models. J Allergy Clin Immunol Pract.

[19] Lieu TA, Capra AM, Quesenberry CP, Mendoza GR, Mazar M (1999). Computer-based models to identify high-risk adults with asthma: is the glass half empty of half full?. J Asthma.

[20] Schatz M, Nakahiro R, Jones CH, Roth RM, Joshua A, Petitti D (2004). Asthma population management: development and validation of a practical 3-level risk stratification scheme. Am J Manag Care.

[21] Grana J, Preston S, McDermott PD, Hanchak NA (1997). The use of administrative data to risk-stratify asthmatic patients. Am J Med Qual.

[22] Forno E, Fuhlbrigge A, Soto-Quirós ME, Avila L, Raby BA, Brehm J, Sylvia JM, Weiss ST, Celedón JC (2010). Risk factors and predictive clinical scores for asthma exacerbations in childhood. Chest.

[23] Chen T, Guestrin C (2016). XGBoost: A scalable tree boosting system. Proceedings of the ACM SIGKDD International Conference on Knowledge Discovery and Data Mining.

[24] Luo G, He S, Stone BL, Nkoy FL, Johnson MD (2020). Developing a model to predict hospital encounters for asthma in asthmatic patients: secondary analysis. JMIR Med Inform.

[25] Luo G, Nau CL, Crawford WW, Schatz M, Zeiger RS, Rozema E, Koebnick C (2020). Developing a predictive model for asthma-related hospital encounters in patients with asthma in a large integrated healthcare system: secondary analysis. JMIR Med Inform.

[26] Luo G, Johnson MD, Nkoy FL, He S, Stone BL (2020). Automatically explaining machine learning prediction results on asthma hospital visits in patients with asthma: secondary analysis. JMIR Med Inform.

[27] Koebnick C, Langer-Gould AM, Gould MK, Chao CR, Iyer RL, Smith N, Chen W, Jacobsen SJ (2012). Sociodemographic characteristics of members of a large, integrated health care system: comparison with US Census Bureau data. Perm J.

[28] Desai JR, Wu P, Nichols GA, Lieu TA, O'Connor PJ (2012). Diabetes and asthma case identification, validation, and representativeness when using electronic health data to construct registries for comparative effectiveness and epidemiologic research. Med Care.

[29] Wakefield DB, Cloutier MM (2006). Modifications to HEDIS and CSTE algorithms improve case recognition of pediatric asthma. Pediatr Pulmonol.

[30] Luo G (2016). Automatically explaining machine learning prediction results: a demonstration on type 2 diabetes risk prediction. Health Inf Sci Syst.

[31] Liu B, Hsu W, Ma Y (1998). Integrating classification and association rule mining. Proceedings of the 4th International Conference on Knowledge Discovery and Data Mining.

[32] Thabtah FA (2007). A review of associative classification mining. Knowledge Eng Review.

[33] Fayyad UM, Irani KB (1993). Multi-interval discretization of continuous-valued attributes for classification learning. Proceedings of the 13th International Joint Conference on Artificial Intelligence.

[34] Molnar C (2020). Interpretable Machine Learning.

[35] Guidotti R, Monreale A, Ruggieri S, Turini F, Giannotti F, Pedreschi D (2019). A survey of methods for explaining black box models. ACM Comput Surv.

[36] Alaa AM, van der Schaar M (2018). Prognostication and risk factors for cystic fibrosis via automated machine learning. Sci Rep.

[37] Alaa AM, van der Schaar M (2018). AutoPrognosis: automated clinical prognostic modeling via Bayesian optimization with structured kernel learning. Proceedings of the 35th International Conference on Machine Learning.

[38] Rudin C, Shaposhnik Y (2019). Globally-consistent rule-based summary-explanations for machine learning models: application to credit-risk evaluation. Proceedings of INFORMS 11th Conference on Information Systems and Technology.

[39] Ribeiro MT, Singh S, Guestrin C (2018). Anchors: high-precision model-agnostic explanations. Proceedings of the 32nd AAAI Conference on Artificial Intelligence.

[40] Liu LL, Forgione DA, Younis MZ (2012). A comparative analysis of the CVP structure of nonprofit teaching and for-profit non-teaching hospitals. J Health Care Finance.

[41] Luo G, Stone BL, Koebnick C, He S, Au DH, Sheng X, Murtaugh MA, Sward KA, Schatz M, Zeiger RS, Davidson GH, Nkoy FL (2019). Using temporal features to provide data-driven clinical early warnings for chronic obstructive pulmonary disease and asthma care management: protocol for a secondary analysis. JMIR Res Protoc.

[42] Luo G (2019). A roadmap for semi-automatically extracting predictive and clinically meaningful temporal features from medical data for predictive modeling. Glob Transit.

